# Ecotoxicological and Interactive Effects of Copper and Chromium on Physiochemical, Ultrastructural, and Molecular Profiling in* Brassica napus* L.

**DOI:** 10.1155/2018/9248123

**Published:** 2018-05-16

**Authors:** Lan Li, Kangni Zhang, Rafaqat A. Gill, Faisal Islam, Muhammad A. Farooq, Jian Wang, Weijun Zhou

**Affiliations:** ^1^Institute of Crop Science and Zhejiang Key Laboratory of Crop Germplasm, Zhejiang University, Hangzhou 310058, China; ^2^Oil Crops Research Institute, Chinese Academy of Agricultural Sciences, Wuhan 430000, China; ^3^Institute of Pure and Applied Biology, Bahauddin Zakariya University, Multan 60000, Pakistan

## Abstract

Heavy metal accumulation causes huge environmental problems, particularly in agricultural ecosystems which have deteriorative effects on the yield and quality of crops. Individual copper (Cu) and chromium (Cr) effects have been investigated extensively in plants; however, co-contamination of Cu and Cr induced stress on* Brassica napus* L. is still unclear. In the present experiment, the interactive effects of Cu and Cr were studied in two* B. napus* cultivars (Zheda 622 and ZS 758). Results showed that the application of Cr was more toxic than Cu, and their combined stress had shown a significant adverse effect on plant growth. Biomass and photosynthetic pigment were decreased remarkably under all metal treatments. Individual treatments of Cu and Cr and their combination cause the accumulation of ROS and lipid peroxidation. Moreover, the activities of antioxidant enzymes and their mRNA transcription levels, such as catalase (CAT), ascorbate peroxidase, glutathione reductase, superoxide dismutase, and peroxidase, were increased, especially when treated with Cr alone or under Cu+Cr combined treatment in both cultivars, except for the CAT activity which was decreased in both leaves and roots of sensitive cultivar Zheda 622 as compared with their respective controls. Additionally, nonenzymatic antioxidants like reduced and oxidized glutathione showed a differential activity pattern in roots and leaves of both cultivars. A more pronounced modification in chloroplast ultrastructure was observed in both cultivars under Cu+Cr treatment followed by Cr and Cu alone treatments. Furthermore, synergistic effects of Cu and Cr were prominent; this may be due to the enhanced metals uptake under combined treatment, which suggests that Cr and Cu interaction is not competitive but is rather additive and genotypic-dependent.

## 1. Introduction

In recent years, mining and agricultural activities have brought about considerable environmental concern in the form of soil contamination by heavy metals, including Cu and Cr that are considered to be detrimental to plant development and human health. At elevated levels, Cu and Cr metals cause serious alterations in the metabolic functions of plants, notably due to the excessive production of reactive oxygen species (ROS). The redox properties of the two metals are involved in the direct generation of ROS via Fenton and Haber–Weiss reaction [1]. Excess accumulations of ROS generally cause disturbances in vital cellular processes like photosynthesis, respiration, and metabolism of important enzymatic functions [2]. Moreover, elevated levels of metals may also cause structural and ultrastructural damage that impairs DNA molecules and hampers the development and productivity of crops [1].

Large amounts of Cu and Cr are mined/produced every year worldwide owing to their uses in various industrial and agricultural activities. Cr mining production continued to increase from 2001, and total atmospheric emissions of chromium have increased at annual growth rates of 8.8% [3, 4]. China was the leading Cr consumer, stainless steel producer, and ferrochromium-producing country in 2015 [4]. In China, the total atmospheric emission of Cr has been approximately 1.92 × 10^5^ tons since 1990, and coal combustion, steel, cast iron, and leather tanning industry are considered as the main sources of pollution in Guangdong, Zhejiang, Jiangsu, Shandong, and Hebei provinces [3]. Cr fugitive emissions from industrial cooling towers and road dust are considered as the most important sources of Cr [5]. Soil contamination by Cu and Cr is increasing due to their natural occurrence in the soils coupled with their intensive industrial uses in the modern era. Although Cr exists in several oxidant states, the hexavalent Cr^6+^ and trivalent Cr^3+^ forms are the most stable. Cr is ranked the seventh most abundant element on Earth [6]. Similarly, Cu is an essential trace element required by plants and other living organisms as a micronutrient. Excess Cu, however, is one of the most harmful pollutants, which affects both terrestrial and aquatic life. Like most of the heavy metals, Cu, as an effluent, is introduced to the environment by anthropogenic means, such as sewage irrigation, mining activities, and intensive use of pesticides and herbicides and inorganic fertilizers [7]. Approximately 3.4 million tons of copper is released to the terrestrial surface every year [8]; thus, it is necessary to study how the crops are affected by copper pollution in response to the presence of other metals in agricultural fields.

Plants can modulate potential oxidative damage caused by different heavy metals [2, 9, 10]. They possess a well-organized network of protective mechanisms, among which the antioxidant system seems to play a major role. Superoxide dismutase (SOD) which converts superoxide radical (O_2_^∙−^) to hydrogen peroxide (H_2_O_2_) is an essential component of the antioxidative system. Catalase (CAT) and peroxidase (POD) in the cytoplasm and other cellular components and ascorbate peroxidase (APX) in the ascorbate-glutathione cycle are also involved in the antioxidative process, reducing H_2_O_2_ to water and oxygen. Additionally, antioxidant compounds, including nonenzymatic molecules such as glutathione and ascorbate [11], are also involved in defense responses under oxidative stress [9].

The use of the crops to manage the polluted soil that contains heavy metals has become an environmentally friendly mode of remediation strategy [12]. The plant species used under heavy metal contaminated soils are assumed to produce higher biomass and possess heavy metal accumulation traits. Many crops have been studied before and Brassicaceae family was found to be a potential source to absorb/accumulate heavy metals from contaminated soils. Among this family,* Brassica napus* L. are widely grown around the world and have been investigated extensively for the remediation of heavy metals [11, 13, 14]. However, planting in heavy metal polluted soils has serious concerns about its overall productivity and grain quality. So, it is necessary to understand the uptake, distribution, and storage of heavy metals within the plant to minimize the risk of metal transfer to the consumer diet. A few investigations have yet reported the interactive effects of Cr^6+^ and Cu^2+^ on different crop plants and their absorption, translocation, and distributive patterns. The present study was, therefore, commenced to examine the Cr^6+^ and Cu^2+^ uptake, translocation, and accumulation pattern in contrasting* B. napus* cultivars and their combined consequences on growth and productivity, chlorophyll synthesis, ROS production, antioxidative defense system, and tolerance mechanisms of* B. napus*. Our findings could provide a more comprehensive understanding of plant tolerance to toxic metals with diverse functions, toxicity thresholds, and competitive properties in metal sensitive and tolerant* B. napus* cultivars.

## 2. Materials and Methods

### 2.1. Plant Material and Growth Conditions

Seeds of two* Brassica napus* L. cultivars [Zheda 622 (ZD 622) and ZS 758] were kindly provided by the College of Agriculture and Biotechnology, Zhejiang University. Healthy and uniform seeds were surface-sterilized in 0.1% NaClO for 15 min, and then the seeds were rinsed for 20 min with distilled water. Fifty seeds were sown in a Petri dish, containing moistened filter paper. The Petri dishes were kept in the dark for 48 h and transferred to a growth chamber with a photoperiod of 16 h day/8 h night, and temperature was set at 22/18°C (day/night), irradiance was 300 *μ*mol m^−2^ s^−1^, and 60–70% relative humidity was used until complete germination. Petri dishes with 30 uniformly germinated seedlings on filter paper and sponge were used as experimental units, in a hydroponic experiment design comprising 4 treatments, based on findings from our previous studies: control, Cu (200 *μ*M), Cr (200 *μ*M), and Cu+Cr (200 *μ*M + 200 *μ*M), with Cu and Cr supplied in the form of CuCl_2_·2H_2_O and K_2_Cr_2_O_7_, respectively. Four replications were used for each treatment. The Hoagland solution was renewed every 4 days, and pH was adjusted to 5.8 ± 0.1 using 1 M NaOH or HCl.

Plant materials were harvested after 10 days of metal exposure. Plants for morphological analyses were divided into shoots and roots, for the measurement of root and shoot length and weight. For dry biomass, plants were placed in an oven at 80°C for 5 days [15]. Samples for physiological and biochemical analysis were frozen in liquid nitrogen and kept in a fridge (−80°C) until use.

### 2.2. Determination of Chlorophyll Pigments and Total Soluble Protein

Leaf chlorophyll pigments were measured according to the method of Porra et al. [16]. The total soluble proteins (TSP) were determined spectrophotometrically according to the method of Bradford [17].

### 2.3. Analysis of Cu and Cr Concentration in Plant Tissue

For the analysis of Cu and Cr content in plant, tissue samples were ashed in a muffle furnace at 550°C for 12 h. Ashed samples were incubated in 1 : 1 HNO_3_ until complete dissolution. Then, the solution was filtered prior to metal analysis. An atomic absorption spectrometer (iCAT-6000-6300, Thermo Scientific, USA) was used to determine the Cu and Cr concentrations.

### 2.4. Determination of Malondialdehyde (MDA) and Reactive Oxygen Species (ROS)

MDA contents in the leaves and roots were determined to estimate the lipid peroxidation of seedlings under each treatment according to the method of Zhou and Leul [18]. For determination of hydrogen peroxide (H_2_O_2_) levels, extraction was performed using fresh leaves and roots (0.5 g) with 5 mL of 0.1% (w/v) trichloroacetic acid (TCA). The slurry was centrifuged at 12000 g for 15 min. The 0.5 mL supernatant was added to a mixture solution containing 0.5 mL of 10 mM potassium phosphate buffer (pH 7) and 1 mL 1 M KI. The absorbance of the mixture was measured at 390 nm. The content of H_2_O_2_ was calculated using a standard curve [19].

The superoxide anion (O_2_^∙−^) levels were determined spectrophotometrically by the method of Jiang and Zhang [20]. Fresh leaves and roots samples (0.5 g) were homogenized in 3 mL of 65 mM potassium phosphate buffer (pH 7.8); the homogenate was then centrifuged at 4°C at 5000 ×g for 10 min. 1 mL of the supernatant was mixed with the reaction solution, which contained 0.9 mL potassium phosphate buffer (65 mM, pH 7.8) and 0.1 mL of hydroxylamine hydrochloride (10 mM), and then the mixture was incubated at 25°C for 20 min. After the incubation, 1 mL of 17 mM sulphanilamide and 7 mM of 1 mL *α*-naphthylamine were added to the mixture for further reaction for 20 min at 25°C. After the reaction, 3 ml of n-butanol was added to the reaction solution and then the mixture was centrifuged for 5 min at a speed of 1500 g. The supernatant was taken and absorbance was read at 530 nm. The generation rate of O_2_^∙−^ was calculated according to a standard curve.

The extracellular hydroxyl radical (^−^OH) was measured following Halliwell et al. [21]. 0.1 g of fresh samples of leaves and roots was homogenized in 1 mL of 10 mM sodium phosphate buffer (pH 7.4) which contained 15 mM 2-deoxy-D-ribose and then incubated for 2 h at 37°C. After incubation, 0.7 mL of the mixture was added to the reaction solution containing 1 mL of glacial acetic acid and 3 mL of 0.5% (w/v) thiobarbituric acid (TBA, 1% stock solution made in 5 mM NaOH). Then, the mixture is put in a water bath at 100°C for 30 min and then the mixture was immediately cooled down on ice to 41°C for 10 min. The absorbance was measured at 550 nm.

### 2.5. Histochemical Analysis

O_2_^∙−^ and H_2_O_2_ production was assayed, respectively, through NBT and 3,3-diaminobenzidine (DAB) staining method as described by Farooq et al. [11]. NBT- and DAB-stained roots were photographed using a digital camera (model Leica, MZ95, Germany), and the presence of brown and blue spots on root tips indicated the physical location of H_2_O_2_ and O_2_^∙−^.

### 2.6. Measurement of Antioxidant Activities

Frozen samples (0.5 g) stored at −80°C were homogenized under liquid nitrogen conditions. Subsequently, the powder samples were transferred to potassium buffer (50 mM, pH 7.0) containing 1 mM EDTA-Na_2_ and 0.5% PVP (w/v). The homogenate was centrifuged at 12000 rpm at 4°C for twenty minutes. The supernatants were stored at −80°C and used for the determination of various antioxidant enzymes. The method of Giannopolitis and Ries [22] was used to determine total SOD (EC 1.15.1.1) activity, based on the ability of SOD to inhibit the photochemical reduction of nitroblue tetrazolium chloride (NBT). 150 *μ*L of the enzyme extract was added to 3 mL of the assay mixture containing 50 mM potassium phosphate buffer (pH 7.8), 0.1 mM EDTA, 13 mM methionine, 75 *μ*M NBT, and 2 *μ*M riboflavin. NBT photoreduction rate was measured at 560 nm after 20 min illumination reaction at 4000 lux of light intensity. One unit of SOD activity was defined as the amount of enzyme that is required to inhibit the reduction of NBT by 50%.

CAT (EC 1.11.1.6) activity was measured with a method based on the determination of the H_2_O_2_ decomposition rate at 240 nm for 1 min [23]. The 3 mL assay mixture contained 50 mM phosphate buffer (pH 7.0), 3.2 mM H_2_O_2_, and 100 *μ*l of enzyme extract. POD (EC 1.11.1.7) activity was determined by guaiacol reduction method as described by Zhou and Leul [24] with some modification. The method of Nakano and Asada [25] helped to assess the activity of ascorbate peroxidase (APX, EC 1.11.1.11). 3 ml assay mixture contained 100 mM phosphate buffer (pH 7), 0.1 mM EDTA-Na_2_, 0.3 mM ascorbic acid, 0.06 mM H_2_O_2_, and 100 *μ*L enzyme extract; absorption was taken at 290 nm for 1 min. To measure GR (EC 1.6.4.2), the assay mixture contained 150 *μ*L extract, 0.5 mM oxidized glutathione (GSSG), and 0.1 mM NADPH in 100 mM sodium phosphate buffer (pH 7.8) added to make a 3 mL assay mixture [26].

The contents of reduced glutathione (GSH) and oxidized glutathione (GSSG) were analyzed according to the method of Law et al. as described by Gill et al. [14] with some modifications. 0.5 g of plant tissues was ground in 5 mL of TCA (10%) solution and centrifuged at 15000 rpm for 15 min. Briefly, 3 mL of the reaction mixture contains 150 *μ*L of enzyme extract, 6 mM 5,5′-dithiobis-2-nitrobenzoic acid (100 *μ*L), 10 U mL^−1^ GR (50 *μ*L), and 0.3 mM NADPH (700 *μ*L) which was measured for total glutathione. For measurement of GSSG, the mixture solution totally contained 120 *μ*L supernatant, 10 *μ*L 2-vinylpyridine, and 20 *μ*L 50% (v/v) triethanolamine. The mixture was shocked for 30 s and then incubated for 25 min at 25°C. Calibration curve was developed using GSSG samples treated exactly as above. The level of GSH for each sample was obtained by subtracting the GSSG level from the total glutathione. All reagents that were used in GSH/GSSG measurement were prepared in 125 mM NaH_2_PO_4_ buffer, containing 6.3 mM EDTA (pH 7.5).

### 2.7. Gene Expression Analysis

Expressions of antioxidative genes were analyzed by quantitative real-time PCR (qRT-PCR). The total RNA of the leaves and roots was extracted using a MiniBEST Plant RNA Extraction Kit (TaKaRa, Japan) which contained gDNA Eraser. PrimeScript™ RT reagent kit was used for cDNA synthesis. Real-time PCR reaction was performed in a final volume of 25 *μ*l using SYBR® Premix Ex Taq II (Takara) according to the manufacturer's instructions. The CFX96TM Real-Time Detection System Software was used to calculate the threshold cycle values, and quantification of mRNA levels was calculated as described by Livak and Schmittgen [27]. Three replicates for each sample were utilized for the real-time PCR analysis. Primers used for qRT-PCR are listed in Table S1.

### 2.8. Ultrastructural Observation of Leaves and Root Tips

For ultrastructural observation, the method of Wang et al. [28] was applied with some modifications. Leaf samples (about one mm^2^) without veins and root samples (about 2-3 mm) were collected. The samples were soaked in 2.5% (v/v) glutaraldehyde in a phosphate buffer (100 mM, pH 7.4). The samples were washed with the same phosphate buffer three times. The washed samples were fixed with 1% osmium tetroxide (OsO_4_) for 1 h. After fixation, samples were washed three times with phosphate buffer. Samples were then dehydrated in an ethanol series (50, 60, 70, 80, 90, 95, and 100%, v/v) for 15 min in each interval and then finally washed in absolute acetone for 20 min. After dehydration, the samples were embedded in Spurr's resin overnight and heated for 9 h at 70°C. Ultrathin sections (80 nm) were used for transmission electron microscopy (TEM 1230EX, JEOL, Japan) examinations.

### 2.9. Statistical Analysis

The analysis of variance (ANOVA) was performed using statistical package SPSS 20 (SPSS, Chicago, IL, USA) for detection of statistically significant differences at *P* < 0.05. The results were expressed as mean ± SD for at least three replicates, and Duncan's multiple range test was carried out for multiple comparisons between treatments.

## 3. Results

### 3.1. Morphological, Chlorophyll, and Soluble Protein Analysis

Morphological changes induced by excess Cu and Cr, measured in terms of the decrease of fresh and dry biomass, shoot height, and root length, are presented in Table S2. The effects of both metals (Cr and Cu) were observed in all the studied morphological parameters between treated plants and controls. In both cultivars (ZD 622 and ZS 758), more pronounced reductions were noted in Cr-treated plants than in their counterparts under Cu treatments, implying a higher toxicity of Cr when applied at equimolar exposure strength. The Cu and Cr combined treatment reduced plant growth comparatively under single Cr or Cu stress. A variation in the extent of Cr- and Cu-induced changes was obvious between the two studied cultivars; higher damage was observed in cultivar ZD 622.

The effects of Cu and Cr were also manifest on chlorophyll accumulation and total soluble protein content in the leaf tissues (Table 1). Similar to morphological changes, Cu and Cr provoked marked decline of chlorophyll and protein content in leaves of both cultivars, signifying a considerable impairment of photosynthetic activity in these cultivars. Further, Cr alone treatment induced significant effects compared with Cu treatment, and the combined supply of these metals showed more significant effects on the loss of chlorophyll and protein in both cultivars ZD 622 and ZS 758. In ZD 622, the sensitive cultivar, a more prominent decline was recorded compared with ZS 758, the less sensitive one.

The ROS and MDA accumulation, commonly observed in stressed plants, was also determined in both root and leaf tissues of treated* B. napus* plants (Tables 2 and 3). In two cultivars (ZD 622 and ZS 758), significant amounts of MDA, H_2_O_2_, O_2_^∙−^, and ^−^OH were accumulated in the root and leaf tissues followed by the exposure to Cr and Cu stress. These metals are free radical generators and the current study revealed a higher rate of ROS formation under Cr alone treatment compared with Cu alone treatment. In fact, higher levels of MDA and ROS were noticed in cultivar ZD 622 than in ZS 758.

### 3.2. Total Cu and Cr Concentration in Plant Tissues

Concentrations of Cu and Cr in shoots and roots of two* B. napus* cultivars exposed to Cu and Cr treatment are presented in Table 4. From the results, it was observed that Cu and Cr accumulation in plants was significantly increased after Cu and Cr treatments, and the contents of Cu and Cr were higher in roots than in shoots under all stress treatments. It was also found that when treated with Cu+Cr, the accumulation of Cr was much higher than Cr alone treatment in both cultivars. In addition, Cu and Cr contents in cultivar ZS 758 were lower compared with ZD 622 under the same treatment, especially in the root.

### 3.3. Histochemical Staining Detection

**Figure 1:**
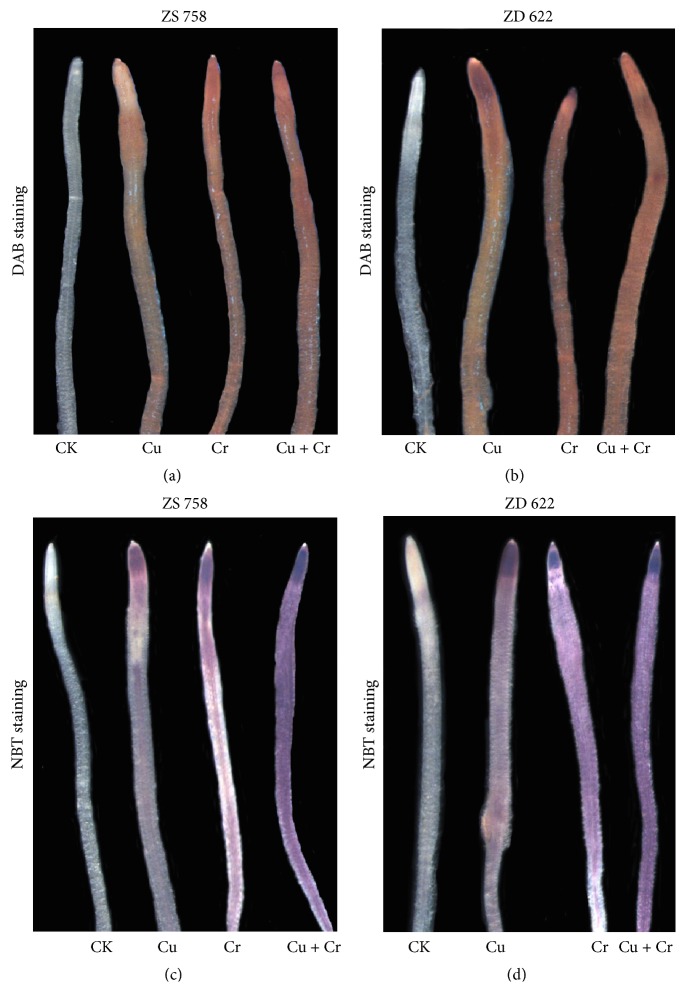
Comparison of ROS (O_2_^∙−^ and H_2_O_2_) production in roots of* Brassica napus *cultivars (ZS 758 and ZD 622) under copper (Cu) and chromium (Cr) alone and combined treatments. Plant roots were stained with NBT (1 g L^−1^) and DAB (1 g L^−1^) immediately after being removed from treatments. The experiment was repeated four times. (a) and (b) represent H_2_O_2_ accumulation stained with DAB, while (c) and (d) represent O_2_^∙−^ staining with NBT.

H_2_O_2_ is a secondary messenger in plant cells and has the ability to trigger various responses. Root tips stained with DAB for H_2_O_2_ showed fewer brown spots under Cu treatment compared with other metal stress treatments. However, root tips applied with Cr and Cu+Cr treatments showed intense dark brown spots in both cultivars. Furthermore, cultivar ZD 622 showed higher accumulation of H_2_O_2_ than ZS 758 under the same treatment levels (Figure 1). Similarly, root tips stained with NBT to detect the accumulation of O_2_^∙−^ are also shown in Figure 1. Blue formazan appeared in all treated root tips except control. Under Cr and Cu+Cr treatments, the root tips showed intense dark coloration in both rapeseed cultivars.

### 3.4. Glutathione Metabolism

**Figure 2:**
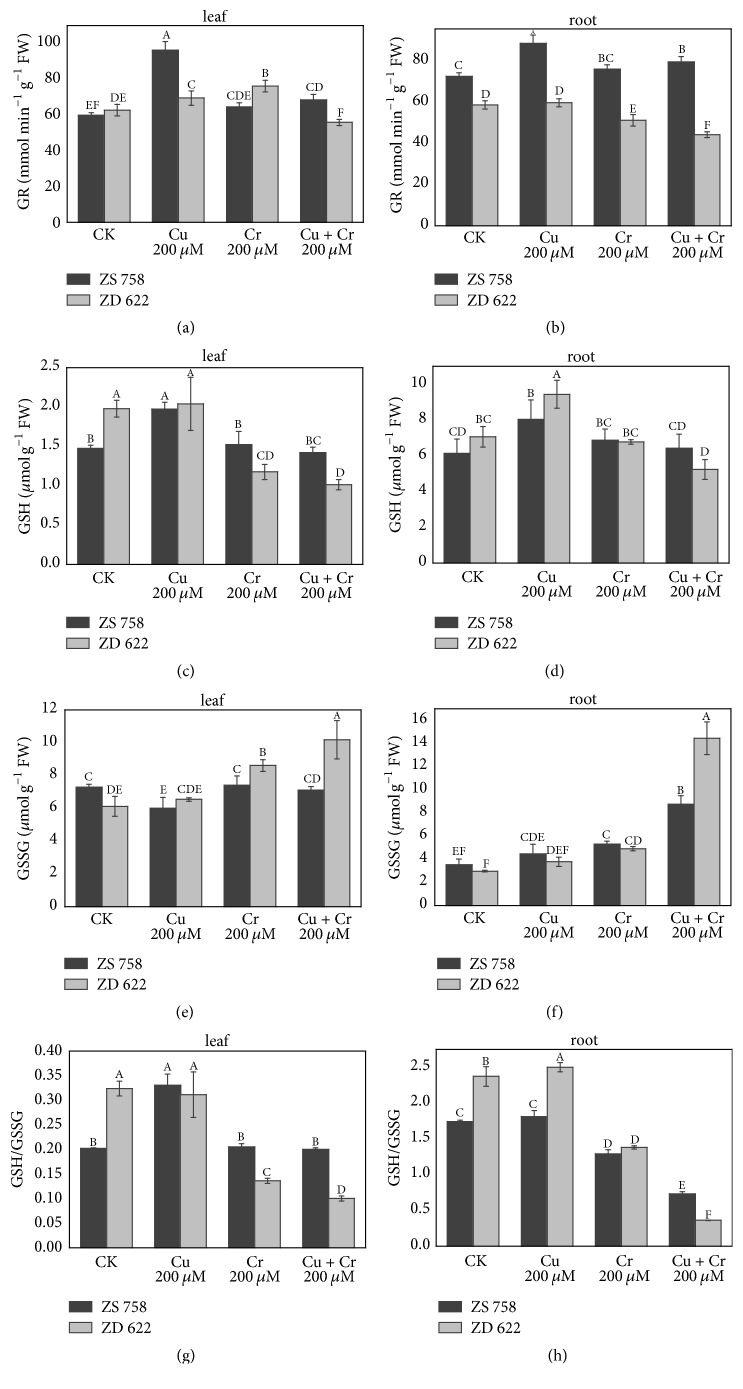
Effect of different treatments of copper (Cu) and chromium (Cr) on the contents of glutathione metabolism in leaves and roots of two* Brassica napus* cultivars. (a, b) Glutathione reductase (GR); (c, d) reduced glutathione (GSH); (e, f) oxidized glutathione (GSSG); (g, h) reduced glutathione/oxidized glutathione (GSH/GSSG) ratio. Each value in the graphs represents the mean with standard deviation of three replications. Means followed by different letters indicate significant difference at *P* < 0.05, according to Duncan's test.

The effects of Cu and Cr on the plant glutathione metabolism are shown in Figure 2. Generally, cultivar ZS 758 maintained higher GR activity in roots while in shoot the GR activity was cultivar- and treatment-dependent. Among the treatments, the highest increase in the GR activity was observed in roots of cultivar ZS 758, while its activity was significantly reduced in ZD 622 under Cu alone treatment in both roots and leaves of* B. napus *plant compared with cultivar ZS 758. Under Cr alone treatment, ZD 622 maintained relatively higher activity of GR in leaves while GR activity in roots was significantly reduced, whereas cultivar ZS 758 showed significantly higher activity of GR in roots compared with leaves where its activity was considerably reduced as compared to cultivar ZD 622. Under combined treatment of Cu and Cr stress, we found that cultivar ZS 758 maintains higher GR activity than ZD 622 in both roots and leaves.

Data show that Cu and Cr stress generally increased the contents of GSH and GSSG in plant tissues. The content of GSH in leaves and roots was significantly increased under Cu alone treatment in both cultivars, except for the leaves of ZD 622, whereas under Cr and combined treatment, GSH activity was downregulated significantly in leaves of ZD 622 compared with its respective control. Conversely, GSH accumulation was increased up to 12% and 5% in the roots of ZS 758 with exposure to Cr and combined stress treatments, respectively. Moreover, in the leaves of ZS 758, no significant change in the accumulation of GSH was observed under Cr and combined stress treatments compared with control, respectively.

Generally, the accumulation of GSSG under metal treatments was high in cultivar ZD 622 compared with ZS 758. In leaves of cultivar ZS 758, no significant change in the GSSG concentration was observed compared with control, while a significant increase in GSSG accumulation was found under individual and combined metal stress conditions in ZD 622. Additionally, the GSSG accumulation in the roots of ZS 758 was increased 1.3-, 1.5-, and 2.5-fold compared with control, with a 1.2-, 1.7-, and 4.9-fold increase in ZD 622 under individual treatment of Cu, Cr, and their combined treatments, respectively. The GSH/GSSG ratio was higher under Cu treatment alone in both cultivars, whereas a significant decrease in GSH/GSSG ratio was recorded under Cu+Cr combined treatment followed by single Cr treatment. Among cultivars, ZS 758 maintained a relatively higher level of GSH/GSSG ratio under heavy metals exposure compared with ZD 622.

### 3.5. Antioxidant Enzymes and Their Gene Expression Levels

**Figure 3:**
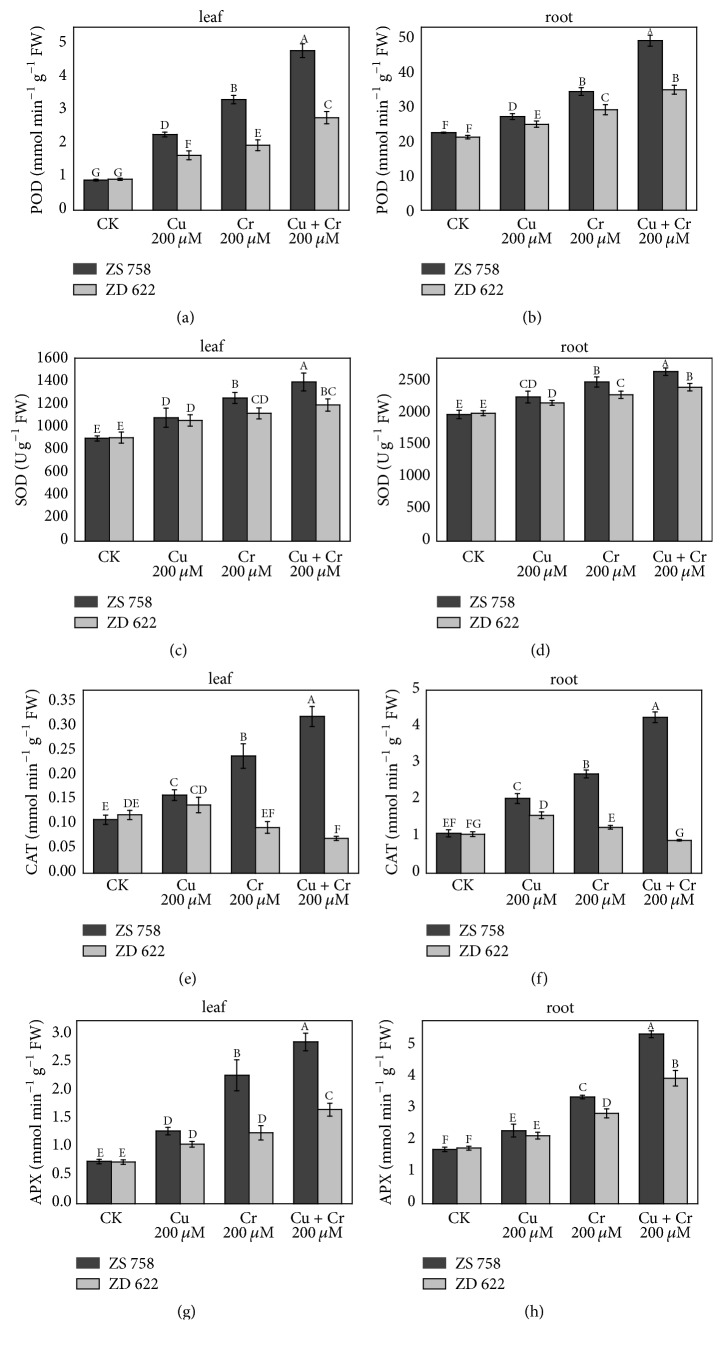
Effect of copper (Cu) and chromium (Cr) treatment on antioxidant enzymes activities in the leaves and roots. (a, b) Guaiacol peroxidase (POD); (c, d) superoxide dismutase (SOD); (e, f) catalase (CAT); (g, h) ascorbate peroxidase (APX). Each value in the graphs shows the mean with standard deviation of three replications. Means followed by different letters indicate significant difference at *P* < 0.05, according to Duncan's test.

*Brassica* plants showed significant alterations in the antioxidant enzyme profiles when they are exposed to the metal stress. Results showed that the patterns of all antioxidant enzyme activities were not similar between each other under the stress conditions. Both Cu and Cr treatments increased the activities of POD and SOD in both leaves and roots of ZS 758 and ZD 622 compared with their respective controls. However, higher activity was noted in ZS 758 than in ZD 622 with the same treatment levels (Figure 3). In Cu treatment, a significant increase was observed in POD and SOD activities in both cultivars, whereas under Cr stress, a higher increase in activities of these two enzymes was observed. But no significant difference was seen between Cr and Cu+Cr treatments in ZD 622 leaf regarding the SOD activity.

Remarkable difference was recorded in CAT activities in two cultivars. Results showed the gradual increase in leaf and root under the all treatments, that is, control, Cu, Cr, and Cu+Cr in ZS 758. But in ZD 622, CAT activity was highest in the Cu treatment in leaf and root, while it was decreased at Cr and Cu+Cr treatments. The APX activity was significantly enhanced after heavy metal treatments. In leaf, it was increased by 72%, 204%, and 283% in ZS 758 and 43%, 70%, and 126% in ZD 622 under Cu, Cr, and Cu+Cr treatments as compared with their respective controls. The variation trend of APX activity in the root was similar to leaf. However, it was increased more remarkably in leaf than in the root of ZS 758.

**Figure 4:**
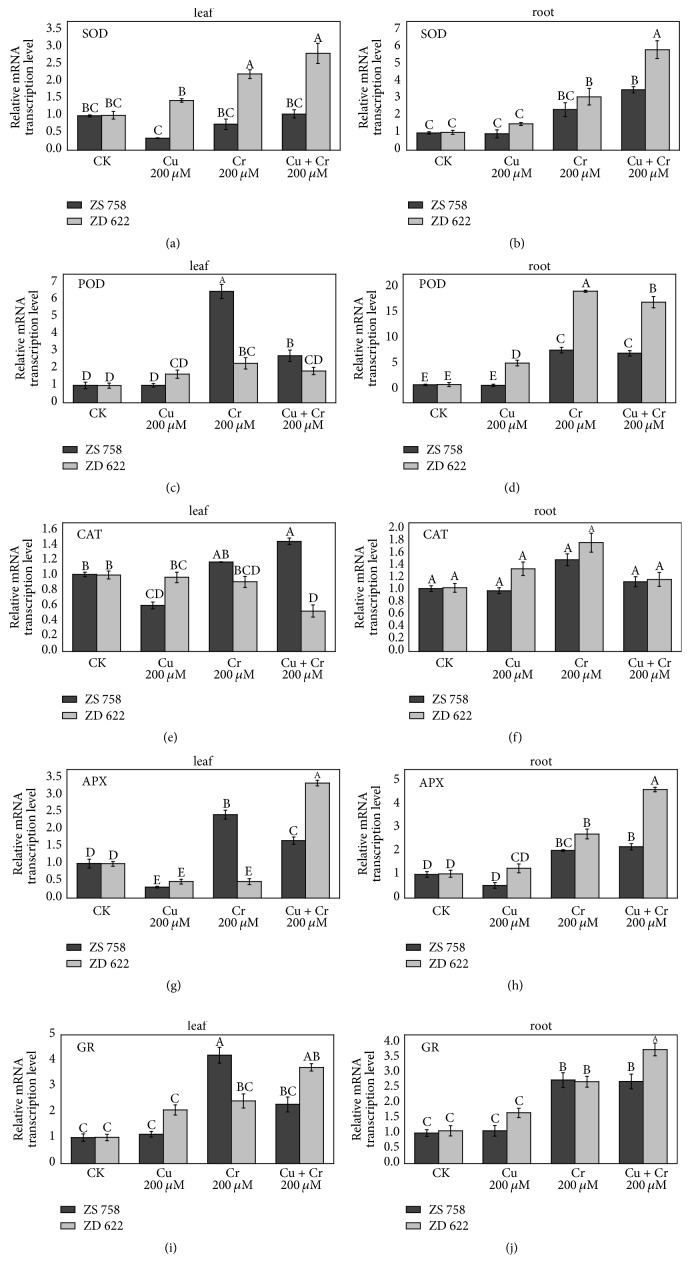
Effect of different treatments on relative genes expression levels of antioxidant enzymes in leaves and roots of ZS 758 and ZD 622. (a, b) Superoxide dismutase (SOD); (c, d) guaiacol peroxidase (POD); (e, f) catalase (CAT); (g, h): ascorbate peroxidase (APX); (i, j) glutathione reductase (GR). Each value in the graphs shows the mean with standard deviation of three replications. Means followed by different letters indicate significant difference at *P* < 0.05 according to Duncan's multiple range test.

The expression levels of antioxidant genes were investigated using qT-PCR analysis as shown in Figure 4. As the results showed, the expression of SOD in ZD 622 was upregulated linearly in both roots and leaves and its expression level was significantly higher in cultivar ZD 622 compared with ZS 758 under all stress treatments. Conversely, expression of SOD in leaves of ZS 758 was downregulated under individual Cu and Cr treatment, while no significant change was noticed under combined stress treatment in leaves. In roots of cultivar ZS 758, SOD expression was significantly upregulated under combined stress treatment compared with control. The POD expression was significantly provoked by Cr alone treatment in leaves of cultivar ZS 758, whereas its transcript abundance was high under Cr alone and combined Cu+Cr treatment in roots of ZD 622. Generally, the expression of POD was much induced in roots compared with leaves of* B. napus* cultivars. CAT expression was nonsignificantly induced in roots of* B. napus* cultivars under individual Cu and Cr treatment, while no change was found in CAT expression under combined treatments of Cu+Cr in roots of both cultivars. In leaves, we found differential activity patterns of CAT under stress treatments, where Cr alone and combined treatments inhibited the expression of CAT in ZD 622 while the opposite was true for ZS 758.

Similarly, APX expression in leaves was inhibited by Cu treatment in both cultivars, while under Cr treatment expression of APX was raised significantly in leaves of cultivar ZS 758. However, under combined treatment of Cu+Cr, APX expression was significantly improved in leaves of ZD 622 compared with control and ZS 758. In roots, the expression of APX was significantly enhanced under Cr alone and combined stress treatment in both cultivars with strong induction in cultivar ZD 622. Under Cu alone treatment, APX expression was inhibited in cultivar ZS 758 while it was nonsignificantly increased in roots of ZD 622. The GR transcript abundance under Cr alone and combined Cu+Cr treatment was significantly higher compared with Cu alone treatment, where nonsignificant increase in GR transcript abundance was observed in roots of both cultivars. In leaves, the highest transcript abundance was found in cultivar ZS 758 under Cr alone treatment, while GR activity of ZS 758 under combined treatment was nonsignificantly induced compared with its respective control. Additionally, under Cu alone treatment, nonsignificant increase in the transcript of GR was noted in shoot and root for both cultivars, but expression level of GR was higher in cultivar ZD 622 compared with ZS 758.

### 3.6. Ultrastructural Changes in Leaf and Root

**Figure 5:**
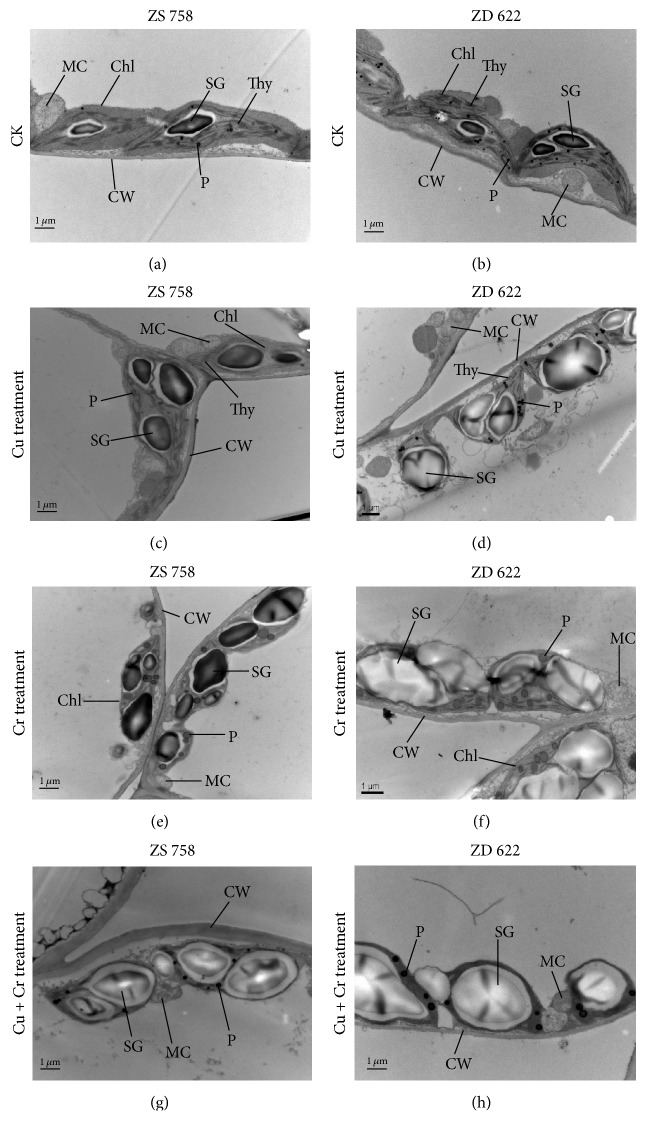
Electron micrographs of leaf mesophyll cells of 10-day grown seedlings of two rapeseed cultivars (ZS 758 and ZD 622) under control (CK) and different treatments (i.e., Cu 200 *μ*M, Cr 200 *μ*M, and Cu 200 *μ*M + Cr 200 *μ*M). (a, b) TEM micrograph of leaf mesophyll cells of ZS 758 and ZD 622 under control (CK) showing well-developed chloroplasts (Chl) with clear thylakoid membrane (Thy), round mitochondria (MC) with distinct cristae inside, starch grains (SG), plastoglobuli (P), and smooth cell wall (CW). (c) TEM micrograph of leaf mesophyll cells of ZS 758 under Cu treatment showing well-developed chloroplasts (Chl) with bigger starch grains (SG), mature mitochondria (MC), smooth cell wall (CW), and plastoglobuli (P). (d) TEM micrograph of leaf mesophyll cells of ZD 622 under Cu treatment showing plastoglobuli (P), swollen chloroplasts (Chl) with large size starch grains (SG), mitochondria (MC), and clear cell wall (CW). (e) TEM micrograph of leaf mesophyll cells of ZS 758 under Cr treatment showing starch grains (SG), large plastoglobuli (P), swollen chloroplasts (Chl), and mitochondria (MC). (f) TEM micrograph of leaf mesophyll cells of ZD 622 under Cr treatment showing increased size and number of plastoglobuli (P) and starch grains (SG), rough cell wall (CW), and disorganized chloroplasts (Chl). (g) TEM micrograph of leaf mesophyll cells of ZS 758 under Cu+Cr treatment showing starch grains (SG), plastoglobuli (P), swollen chloroplasts (Chl), mitochondria (MC), and thick cell wall (CW). (h) TEM micrograph of leaf mesophyll cells of ZD 622 under Cu+Cr treatment showing increased size plastoglobuli (P) and huge starch grains (SG), rough cell wall (CW), disorganized chloroplasts (Chl), and mitochondria (MC) with fewer cristae inside.

The ultrastructural changes in leaf mesophyll and root tip cells of two rapeseed cultivars under control and heavy metal treatments are shown in Figure 5. The structures of leaf mesophyll cells were changed under heavy metal treatments (Figure 5). In the leaf mesophyll cells of control plant, intact and smooth cell wall was observed. In addition, a well-developed chloroplast structure with dense grana and clear thylakoids membranes was observed in the micrographs of control plants. Moreover, mature and round-shaped mitochondria with apparent cristae were also noticed in the micrographs. Under the individual Cu treatment, there was no significant change in ultrastructure of ZS 758 except that the starch grains became bigger than control. However, chloroplasts with swollen shape which contained large starch grains and more plastoglobuli were found in ZD 622. More pronounced modifications in ultrastructure were observed in both cultivars under Cr and Cu+Cr treatments. Under these two treatments, plastoglobuli became bigger and increase in numbers and size, and large starch grains were observed, especially when treated with Cu+Cr in ZD 622, which almost occupied 90% of the chloroplasts, thus resulting in disorganization of thylakoids. Under Cu+Cr treatment, mitochondria were severely damaged.

**Figure 6:**
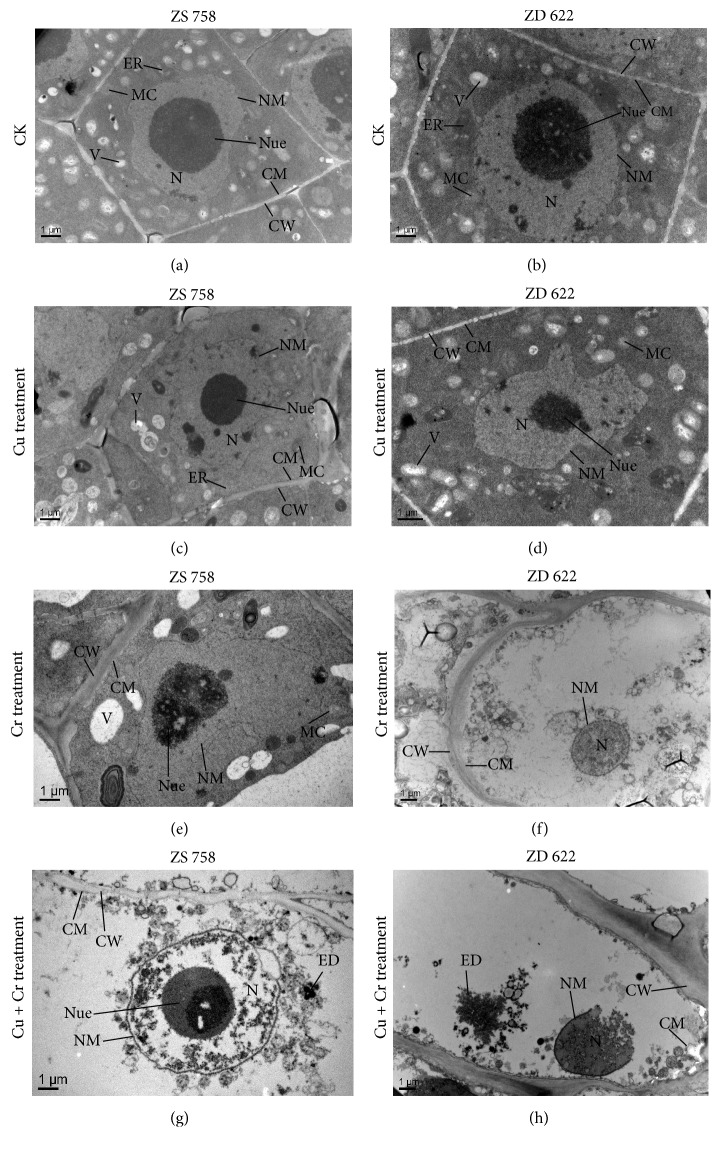
Electron micrographs of root tip cells of 10-day grown seedlings of two rapeseed cultivars (ZS 758 and ZD 622) under control (CK) and treatment (Cu 200 *μ*M, Cr 200 *μ*M, and Cu 200 *μ*M + Cr 200 *μ*M). (a, b) TEM micrograph of root tips of ZS 758 and ZD 622 under control showing large sized nucleus (N) with a round nucleolus (Nue) and well-shaped nuclear membrane (NM), clear cell wall (CW) and cell membrane (CM), oval shape mitochondria (MC), and endoplasmic reticulum (ER). (c) TEM micrograph of root tips of ZS 758 under Cu treatment showing clear cell wall (CW) and cell membrane (CM), round-shaped nucleolus (Nue) and well-developed nucleus (N), and oval shape mitochondria (MC). (d) TEM micrograph of root tips of ZD 622 under Cu treatment showing clear cell wall (CW) and cell membrane (CM), distorted nucleolus (Nue), and oval shape mitochondria (MC). (e) TEM micrograph of root tips of ZS 758 under Cr treatment showing rough cell wall (CW), scattered nucleolus (Nue), and bigger vacuole (V). (f) TEM micrograph of root tips of ZD 622 under Cr treatment showing rough cell wall (CW) and cell membrane (CM) and reputed nucleolus (Nue). (g) TEM micrograph of root tips of ZS 758 under Cu+Cr treatment showing undeveloped nucleus (N) with a roundish nucleolus (Nue), rough cell wall (CW) and cell membrane (CM), and electron dense granules (ED). (h) TEM micrograph of root tips of ZD 622 under Cu+Cr treatment showing thick and rough cell wall (CW), ruptured nucleus (N) and broken nuclear membrane (NM), and electron dense granules (ED).

The TEM micrographs of root cells of ZS 758 and ZD 622 under all treatments are shown in Figure 6. At the control treatment, the root tip cells showed regular shape with clear cell wall. A large size cell nucleus with a well-developed nucleolus and nuclear membrane was noticed in the cell. In addition, both cultivars presented many mature and typical oval shaped mitochondria. The shapes of root tip cells of two cultivars were almost normal, but the cell nucleus was distorted to some extent under Cu treatment, especially in ZD 622. At Cr treatment, cell ultrastructure was damaged severely as compared with Cu, especially in ZD 622. Nucleolus was broken and no mitochondrion was observed in ZD 622 under Cr treatment. Correspondingly, vacuoles became bigger in ZS 758. Furthermore, when plants were treated with Cu and Cr combined treatment, organelles were mostly damaged and vacuolation was observed in both cultivars. No cytoplasm was observed in ZD 622 root tip cell, and plasmolysis also occurred in ZD 622. At this level, cell wall became thick and rough. Additionally, apparent electron dense granules were observed in root of both cultivars.

## 4. Discussion

In the present investigation, we have investigated that Cu and Cr induced oxidative stresses and antioxidant responses of two* B. napus* cultivars under individual and combined application of Cr and Cu. Copper is an essential element for plant growth while, to date, no obvious role of Cr is discovered in plants. However, excess concentration of these metals can affect the plant growth as we found in this study. Earlier, Mwamba et al. [29] and Gill et al. [14] found similar results of growth retardation in* B. napus* plants under Cu and Cr stress conditions, respectively. Here, we also studied the combined effect of Cu and Cr stress on the growth and physiology of* B. napus* plants and found that a combined application of Cu+Cr proved to be more toxic than Cr followed by Cu alone treatments in both cultivars. The decreasing trend of plant biomass production in cultivar ZD 622 was more pronounced as compared to ZS 758, under individual and combined stress treatments. The decrease of plant growth under Cr stress might be due to the transport of Cr with water and nutrients to the aerial part that has a direct impact on cellular metabolism which was further contributing to the reduction of plant growth [2]. On the other hand, Cr affects the net photosynthetic rate because of the inhibition of photosynthetic pigments like Chl* a* and* b,* thus resulting in the decline of dry biomass. Vajpayee et al. [30] found that an essential enzyme, *δ*-aminolaevulinic acid dehydrate, in biosynthesis of chlorophyll was degraded when plants were exposed to Cr, which led to a decrease of photosynthetic pigments. Furthermore, Cu and Cr induced reduction in chlorophyll content and chlorosis might be due to the reduction of Fe availability to the leaves and negative effects of Cr on chlorophyll metabolism. These findings are in line with our results, for instance, reduced chlorophyll concentration and biomass accumulation (Tables 1 and S2). On the other hand, excess Cu exposure to the plant also decreases the biomass production and chlorophyll contents, but reduction in these traits was more pronounced under Cr treatments, which might be attributed to the essential role of Cu in plant growth.

Being a redox-active metal, Cu can generate reactive oxygen species (ROS) via the Haber–Weiss and Fenton reactions that can impair cellular components (DNA, RNA, proteins, amino acids, and membrane lipids), impede cellular transport processes, and alter the concentration of essential metabolites. The increased ROS accumulation was found under Cu and Cr treatments as indicated by enhanced accumulation of H_2_O_2_, O_2_^∙−^, and ^−^OH contents. Generally,* B. napus* plants grown under Cr treatment experienced higher oxidative stress, which is strongly correlated with the enhanced accumulation of ROS (H_2_O_2_, O_2_^∙−^, and ^−^OH) compared to the individual Cu treatment (Tables 2 and 3). Furthermore, ROS contents were also increased significantly when plants were exposed to individual Cu treatment (Tables 2 and 3), which is supported by previous studies conducted by Feigl et al. [31] in which a significant ROS level was produced in both* Brassica* species treated with Cu. Besides this, ROS concentration increased further under combined treatment of Cu and Cr, which is also in line with the previous studies, where the combined treatment of Cr or Cu with other metals proved to be more toxic than their alone treatments [32, 33]. Additionally, the accumulation of ROS in different stress treatments was correlated with the results of histochemical staining of NBT (O_2_^∙−^) and DAB (H_2_O_2_) (Figure 1).

Heavy metal toxicity also enhances the production of lipid peroxidation via generation of free radicals. The quantification of MDA (a byproduct of lipid peroxidation) gives an estimate of the actual effect of free radicals on cell membrane. Our results showed increased H_2_O_2_ production concomitant with lipid peroxidation in Cu- or Cr-stressed plants. In our study, Cu and Cr stress induced oxidative stress through the generation of ROS in both* Brassica* cultivars; however, the damage was more apparent in ZD 622 than in ZS 758, which shows that ZS 758 is better in scavenging ROS to withstand Cu and Cr toxicity.

In order to detoxify the overproduced ROS under stress conditions, plants have evolved a complex antioxidant defense system that consists of enzymatic and nonenzymatic antioxidants. Under metal stress conditions, SOD mainly scavenges the superoxide radical (O_2_^∙−^) by disproportionate reaction and then converts it into H_2_O_2_ [34]. Moreover, H_2_O_2_ generated by SOD is destroyed further by CAT, POD, and APX. Hence, the common role of SOD and CAT is very important to alleviate the hazardous effects caused by ROS, because the CAT, POD, and APX generally act as the catalyzer to digest/break down the H_2_O_2_ produced by SOD. Results show that activity of SOD was increased when plants were exposed to Cu or Cr alone or combined stress in both roots and shoots of* B. napus* cultivars. However, plants under Cr and combined metal treatment showed a substantial increase in activity of SOD, which is correlated with H_2_O_2_ production in* B. napus* cultivars. The Cr and combined stress treatment decreased CAT activity in cultivar ZD 622 compared to ZS 758, which may be the reason why ZD 622 plants showed reduced growth and enhanced oxidative stress under Cr stress and combined metal treatments than Cu alone treatment in* B. napus* plants. Previously, several authors have also observed the decrease of CAT activity, which leads to the higher oxidative stress [2]. CAT has a higher turnover rate for converting H_2_O_2_ to H_2_O and O_2_ than other enzymes. Furthermore, activities of APX and POD were increased significantly in cultivar ZS 758 when compared with ZD 622 under Cr alone and Cu+Cr combined exposure, which may suggest superior antioxidant and scavenging ability of cultivar ZS 758. Liu et al. [2] found that when seedlings of* Amaranthus viridis* L. were treated with Cr^6+^ at 10^-5 ^M and 10^-4 ^M concentrations, POD activity was significantly increased. In another study, Zaheer et al. [35] found that both APX and POD in the roots and leaves were significantly increased under Cu treatment in* B. napus* L.

Besides this, transcript analysis of antioxidant enzyme genes suggests that posttranscriptional and translational modifications are involved and act as the major regulators of enzyme stability and activity [36]. Previously, various studies demonstrated a similar pattern between enzyme transcript and its activity. Usually, enhanced antioxidant enzyme activities are considered as an adaptive strategy, which mainly depends upon the type of genotype and severity of stress. However, a recent study by Fidalgo et al. [37] found no parallel trend between transcript accumulation and enzyme activity under the Cu stress in* Solanum nigrum* L. plants. Similarly, Romero-Puertas et al. [38] and Soares et al. [36] also observed similar results in peas and* S. nigrum* under Cu and Ni stress. Taken together, the results showed that cultivar ZS 758 has comparatively better scavenging mechanism to detoxify the ROS produced by individual Cu and Cr or combined stress treatments.

The glutathione-ascorbate cycle plays a vital role in maintaining cellular redox potential and protection of cell organelles from toxicity induced by ROS under stressful conditions [1]. The cellular ratio of reduced to oxidized glutathione is vital in maintaining a cellular redox state under environmental stress conditions. Glutathione plays a key role in the tolerance and defense against heavy metals-mediated oxidative damage by taking part in various physiological and biochemical processes such as modulation of thiol-disulphide status, reduction of peroxides, and free radical scavenging [10, 34]. In the present investigation, application of metals modulated the GSH and GSSG accumulation and GSH/GSSG ratios under individual and combined stress treatments. Increased oxidation of GSH in ZD 622 may be due to the overproduction of ROS, which may cause reduced GSH/GSSG ratio and higher GSSG content, because GSH is an efficient ROS scavenger which directly scavenges the ^−^OH and O_2_^∙−^ and thus protects the enzyme thiol groups. Similar findings were also reported by Rahman et al. [39] under metal stress conditions. The GR recycles the GSSG to GSH and helps the plants to keep a higher ratio of GSH/GSSG (Figure 2) and plays a critical role in the maintenance of cellular redox state and signal transmission under stress conditions. We found a significant increase in GR activity under combined and individual metal treatments in ZS 758 compared with ZD 622, which correlates with increased GSH levels under stress treatment. In addition, transcript level of GR was also increased significantly under all treatments (Figure 4). High levels of GR activity and glutathione production in ZS 758 compared with ZD 622 indicate that the active involvement of GR and GSH can prevent oxidative stress [14, 40].

As a vital indicator of reversible and irreversible changes in the metabolism under various external stresses, the TSP content in plant leaves and roots was investigated in the study which showed negative correlation under metal stress conditions (Table 1). The TSP content decreased under Cu or Cr stress, and the effect of the two metals in cultivar ZD 622 was more pronounced than ZS 758. The present results also showed that the effect of Cr on TSP degradation was stronger than Cu. These results are supported by previous studies in cauliflower (*Brassica oleracea* L. var.* Botrytis* cv. Maghi) under Cr and Cu stress [41] and in duckweed* (Lemna minor)* treated with Cu and Cd [42]. The inhibitory effect of metals on protein synthesis may be due to the increased protein hydrolysis by catalytic activity and severe oxidative stress imposed under metals stress conditions [43]. Soluble proteins were also assumed to be involved in metal binding and may play a critical role in heavy metal tolerance of plants, which might be the reason why TSP concentration in sensitive cultivar ZD 622 was less than in the tolerant cultivar ZS 758.

In this present investigation, we observed apparent alteration of fine structures in mesophyll cells. Moreover, disorganized chloroplasts with a poorly developed membrane system, large size starch grains, increased number of plastoglobuli, swollen mitochondria, and damaged cell wall were observed in mesophyll cells under metal treatments, which indicates that cells were damaged to some extent, and these results were consistent with the observation of Speranza et al. [44] and Appenroth et al. [45] under Cr application. Additionally, starch grain accumulation under combined treatments might be related to the reduced movement of sucrose transport from source to sink, while enhanced accumulation of plastoglobuli under Cr treatment alone may be originated from the lipid-soluble degradation products from the thylakoid membranes [46]. In an existing study, we also found abundance of plastoglobuli in ZD 622 under Cr treatment followed by combined stress and Cu alone treatment in both cultivars (Figure 5). Indeed, previous studies have also concluded that size or numbers of plastoglobuli were increased under abiotic stress with different heavy metals [47].

Moreover, modifications in the ultrastructure of root tips were also observed in this study. A distorted or broken cell nucleus, damaged organelles, rough cell wall, and large size vacuoles were observed under metals stress conditions. In previous studies, similar results were observed in* B. napus* under arsenic treatments [11]. The phenomenon of enlarged size vacuoles is also common when plants are exposed to environmental stressors [48] which might be correlated with the cell detoxification and tolerance against metal stresses, because vacuolization can sequester metal ions at a specific location and prevent their circulation. However, the damaging effect of metals on ZS 758 was significantly lower than cultivar ZD 622. Since the structures of organelles in ZS 758 were more integrated, this further suggests that ZS 758 is a more metal tolerant cultivar.

Previously, different authors have reported that Cu and Cr showed competitive behavior when applied with some other elements like nickel and phosphorus [49, 50]. However, in this study, application of individual treatment of Cu enhanced the slight accumulation of Cr in leaves with strong elevation in the roots. Similarly, individual treatment of Cr enhanced the accumulation of Cu with three times more in the roots of sensitive cultivar compared with the control. On the other hand, under combined treatment of Cr and Cu, Cr accumulation was significantly enhanced in root and shoot of the sensitive cultivar, while in resistant cultivar Cu and Cr accumulation was like individual treatments (Table 4). This shows that Cr and Cu interaction is not competitive but is rather additive and genotypic-dependent. This could explain the higher accumulation of Cu and Cr and reduced plant growth in sensitive cultivar compared with resistant cultivar under combined metals treatment.

## 5. Conclusions

The present study demonstrates that the heavy metals could significantly affect the physiological processes of two* B. napus* cultivars. Both Cu and Cr toxicity alone and their combined treatments reduced biomass and photosynthetic pigments of plants. Data show that strong oxidative stress was induced under the metal treatments as indicated by the significant increase in MDA and ROS contents, and Cr caused more toxicity than Cu. Correspondingly, activities of enzymatic and nonenzymatic antioxidants and gene expression of SOD, POD, APX, and GR were considerably increased, which were beneficial to alleviate the oxidative stress. Additionally, damage caused by metals on mesophyll cells and root tips was more prominent in cultivar ZD 622 than in ZS 758 as found physically from TEM analysis. Taken together, the two cultivars have different capabilities to cope with heavy metal stress and ZS 758 was more tolerant when exposed to Cr and Cu or their combined application. Furthermore, co-contamination of Cu and Cr resulted in a higher accumulation of metals, thus suggesting a synergistic or additive response. This shows that uptake of one metal is affected by the presence of other metals. On the basis of this experiment, we propose that cultivar ZS 758 could be used for the phytoextraction of metals in Cu or Cr contaminated soils. However, further studies using real soil media are required, in order to clarify the phytotoxicological mechanism caused by these metals.

## Figures and Tables

**Table 1:** Effects of copper (Cu) and chromium (Cr) on chlorophyll (mg g^−1^ FW) and total soluble protein (TSP) content (mg g^−1^ FW) in two *Brassica napus* cultivars under different metal treatments.

Cultivar	Treatment	Chl a	Chl b	Total Chl	Leaf TSP	Root TSP
ZS 758	CK	0.343 ± 0.017^a^	0.282 ± 0.015^a^	0.625 ± 0.002^a^	15.78 ± 0.46^b^	6.64 ± 0.47^a^
	Cu 200 *μ*M	0.250 ± 0.018^b^	0.190 ± 0.020^b^	0.441 ± 0.037^b^	13.20 ± 0.08^c^	4.84 ± 0.04^b^
	Cr 200 *μ*M	0.191 ± 0.020^c^	0.141 ± 0.011^c^	0.332 ± 0.009^c^	11.28 ± 0.49^d^	3.77 ± 0.46^c^
	Cu 200 *μ*M + Cr 200 *μ*M	0.131 ± 0.008^d^	0.091 ± 0.006^d^	0.221 ± 0.014^d^	9.63 ± 0.18^e^	3.23 ± 0.21^d^

ZD 622	CK	0.337 ± 0.024^a^	0.293 ± 0.029^a^	0.630 ± 0.052^a^	16.65 ± 0.54^a^	6.58 ± 0.23^a^
	Cu 200 *μ*M	0.206 ± 0.025^c^	0.152 ± 0.017^c^	0.358 ± 0.042^c^	13.02 ± 0.88^c^	4.93 ± 0.27^b^
	Cr 200 *μ*M	0.150 ± 0.009^d^	0.080 ± 0.009^d^	0.230 ± 0.018^d^	10.47 ± 0.34^d^	3.77 ± 0.14^c^
	Cu 200 *μ*M + Cr 200 *μ*M	0.081 ± 0.006^e^	0.034 ± 0.003^e^	0.115 ± 0.009^e^	8.35 ± 0.33^f^	2.52 ± 0.27^e^

Data are the means of three replicates (mean ± SD). Values followed by different letters indicate significant differences followed by Duncan's multiple range test (*P* < 0.05) for each cultivar at different metal treatments.

**Table 2:** Effects of copper (Cu) and chromium (Cr) on MDA (nmol g^−1^ FW), hydrogen peroxide (H_2_O_2_) (*μ*mol g^−1^ FW), superoxide radical (O_2_^∙−^) (nmol min^−1^ g^−1^ FW), and hydroxyl radical (^−^OH) (*μ*mol g^−1^ FW) in the leaves of two *Brassica napus* cultivars under different metal treatments.

Cultivar	Treatment	Leaf MDA	Leaf ^−^OH	Leaf H_2_O_2_	Leaf O_2_^∙−^
ZS 758	$\overset{\;}{CK}$	6.02 ± 0.21^e^	1.88 ± 0.10^g^	1.57 ± 0.06^f^	0.013 ± 0.0035^g^
	Cu 200 *μ*M	11.20 ± 0.71^d^	10.41 ± 0.88^f^	51.97 ± 2.06^e^	0.526 ± 0.0075^f^
	Cr 200 *μ*M	17.81 ± 1.18^c^	19.43 ± 0.52^d^	71.05 ± 1.80^c^	0.770 ± 0.0280^e^
	Cu 200 *μ*M + Cr 200 *μ*M	25.95 ± 1.08^b^	25.92 ± 1.10^c^	96.19 ± 4.22^b^	0.945 ± 0.0305^c^

ZD 622	$\overset{\;}{CK}$	5.93 ± 0.12^e^	1.99 ± 0.11^g^	1.66 ± 0.06^f^	0.022 ± 0.0046^g^
	Cu 200 *μ*M	16.83 ± 0.81^c^	16.74 ± 0.69^e^	65.50 ± 1.98^d^	0.882 ± 0.0160^d^
	Cr 200 *μ*M	25.44 ± 2.11^b^	28.67 ± 1.13^b^	96.61 ± 3.14^b^	1.127 ± 0.0410^b^
	Cu 200 *μ*M + Cr 200 *μ*M	35.26 ± 1.85^a^	46.96 ± 2.07^a^	145.53 ± 4.89^a^	1.384 ± 0.0190^a^

Data are the means of three replicates (mean ± SD). Values followed by different letters indicate significant differences followed by Duncan's multiple range test (*P* < 0.05) for each cultivar at different metal treatments.

**Table 3:** Effects of copper (Cu) and chromium (Cr) on MDA (nmol g^−1^ FW), hydrogen peroxide (H_2_O_2_) (*μ*mol g^−1^ FW), superoxide radical (O_2_^∙−^) (nmol min^−1^ g^−1^ FW), and hydroxyl radical (^−^OH) (*μ*mol g^−1^ FW) in the roots of two *Brassica napus* cultivars under different metal treatments.

Cultivar	Treatment	Root MDA	Root ^−^OH	Root H_2_O_2_	Root O_2_^∙−^
ZS 758	$\overset{\;}{CK}$	1.45 ± 0.14^f^	1.46 ± 0.07^f^	1.14 ± 0.07^g^	0.524 ± 0.0410^g^
	Cu 200 *μ*M	22.53 ± 1.17^e^	27.45 ± 1.07^e^	24.12 ± 2.23^f^	1.043 ± 0.1031^f^
	Cr 200 *μ*M	34.65 ± 0.83^d^	40.27 ± 2.20^d^	44.17 ± 1.85^d^	1.487 ± 0.0225^d^
	Cu 200 *μ*M + Cr 200 *μ*M	45.78 ± 1.16^c^	52.54 ± 1.58^c^	62.94 ± 2.66^c^	1.782 ± 0.0517^c^

ZD 622	$\overset{\;}{CK}$	1.48 ± 0.13^f^	1.48 ± 0.08^f^	1.06 ± 0.06^g^	0.543 ± 0.0270^g^
	Cu 200 *μ*M	36.60 ± 1.46^d^	39.73 ± 1.09^d^	35.69 ± 2.34^e^	1.348 ± 0.0240^e^
	Cr 200 *μ*M	53.07 ± 1.23^b^	56.97 ± 3.61^b^	69.72 ± 2.08^b^	1.943 ± 0.1009^b^
	Cu 200 *μ*M + Cr 200 *μ*M	72.72 ± 2.12^a^	73.68 ± 2.57^a^	92.82 ± 3.39^a^	2.665 ± 0.0705^a^

Data are the means of three replicates (mean ± SD). Values followed by different letters indicate significant differences followed by Duncan's multiple range test (*P* < 0.05) for each cultivar at different metal treatments.

**Table 4:** Copper (Cu) and chromium (Cr) accumulation (mg kg^−1^ FW) in shoots and roots of two *Brassica napus* cultivars.

Cultivar	Treatment	Total Cu content		Total Cr content	
		Shoot	Root	Shoot	Root
ZS 758	CK	13.53 ± 1.29^d^	33.35 ± 4.13^d^	0.46 ± 0.08^c^	5.39 ± 0.57^c^
	Cu 200 *μ*M	21.38 ± 1.44^a^	214.86 ± 30.05^b^	0.43 ± 0.08^c^	11.57 ± 1.3^c^
	Cr 200 *μ*M	14.44 ± 1.29^cd^	71.37 ± 5.43^c^	12.91 ± 2.42^b^	202.51 ± 26.78^ab^
	Cu 200 *μ*M + Cr 200 *μ*M	19.03 ± 0.99^b^	204.62 ± 15.61^b^	26.59 ± 4.83^a^	205.19 ± 38.66^ab^

ZD 622	CK	14.57 ± 0.77^cd^	25.62 ± 3.12^d^	0.52 ± 0.14^c^	6.67 ± 0.36^c^
	Cu 200 *μ*M	18.59 ± 1.63^b^	231.78 ± 26.36^b^	0.72 ± 0.09^c^	16.34 ± 1.24^c^
	Cr 200 *μ*M	16.11 ± 0.73^c^	75.50 ± 8.02^c^	15.4 ± 0.99^b^	189.38 ± 19.74^b^
	Cu 200 *μ*M + Cr 200 *μ*M	18.89 ± 1.02^b^	294.45 ± 8.86^a^	28.29 ± 1.98^a^	228.45 ± 7.53^a^

Data are the means of three replicates (mean ± SD). Values followed by different letters indicate significant differences followed by Duncan's multiple range test (*P* < 0.05) for each cultivar at different metal treatments.

## Supplementary Materials

Supplementary Materials Table S1: primers used for qRT-PCR. Table S2: Effects of copper (Cu) and chromium (Cr) on shoot height (mm), root length (mm), and leaf, stem, and root biomass (g) in two* Brassica napus* cultivars under different metal treatments.
