# Supplementary material for: Ecotoxicological and Interactive Effects of Copper and Chromium on Physiochemical, Ultrastructural, and Molecular Profiling in Brassica napus L
Source: Biomed Res Int. 2018 May 16;2018:9248123. doi: 10.1155/2018/9248123 (PMC5977033; doi:10.1155/2018/9248123)
Supplement: Supplementary Materials — Table S1: primers used for qRT-PCR. Table S2: Effects of copper (Cu) and chromium (Cr) on shoot height (mm), root length (mm), and leaf, stem, and root biomass (g) in two Brassica napus cultivars under different metal treatments. [file 9248123.f1.pdf]

Supplementary Material

TABLE S1: Primers used for qRT-PCR.

Primer name	From 5' to 3'
SOD F	ACGGTGTGACCACTGTGACT
SOD R	GCACCGTGTTGTTTACCATC
POD F	ATGTTTCGTGCGTCTCTGTC
POD R	TACGAGGGTCCGATCTTAGC
CAT F	TCGCCATGCTGAGAAGTATC
CAT R	TCTCCAGGCTCCTTGAAGTT
APX F	ATGAGGTTTGACGGTGAGC
APX R	CAGCATGGGAGATGGTAGG
GR F	AAGCTGGAGCTGTGAAGGTT
GR R	AGACAGTGTTCGCAAAGCAG
Actin F	TTGGGATGGACCAGAAGG
Actin R	TCAGGAGCAATACGGAGC

TABLE S2: Effects of copper (Cu) and chromium (Cr) on shoot height (mm), root length (mm), and leaf, stem, and root biomass (g) in two *Brassica napus* cultivars under different metal treatments.

Cultivar	Treatment	Leaf FW	Stem FW	Root FW	Leaf DW	Stem DW	Root DW	Shoot height	Root length
ZS 758	CK	2.31±0.095a	0.56±0.050a	0.44±0.045a	0.1436±0.0045a	0.0284±0.0005a	0.0356±0.0007a	32.50±1.87a	33.77±1.06a
	Cu 200µM	2.07±0.075b	0.44±0.040b	0.34±0.050b	0.1111±0.0070c	0.0240±0.0006b	0.0333±0.0016a	24.43±1.40b	29.60±0.89b
	Cr 200µM	1.78±0.121c	0.35±0.035c	0.25±0.040c	0.0909±0.0071d	0.0218±0.0015bc	0.0262±0.0025b	20.80±0.82c	25.27±0.91c
	Cu 200µM + Cr 200µM	1.47±0.090d	0.25±0.025d	0.20±0.055c	0.0732±0.0028e	0.0186±0.0010c	0.0170±0.0018d	17.13±0.42d	22.40±0.80d
ZD 622	CK	2.28±0.105a	0.57±0.035a	0.43±0.055a	0.1281±0.0079b	0.0301±0.0057a	0.0341±0.0039a	30.97±1.10a	34.20±1.40a
	Cu 200µM	1.83±0.110c	0.39±0.060bc	0.27±0.060bc	0.0940±0.0019d	0.0238±0.0005b	0.0270±0.0010b	21.27±1.01c	26.40±1.10c
	Cr 200µM	1.42±0.090d	0.25±0.040d	0.22±0.030c	0.0705±0.0022e	0.0211±0.0011bc	0.0221±0.0009c	17.17±0.70d	19.87±0.85e
	Cu 200µM +Cr 200µM	1.15±0.060e	0.15±0.035e	0.12±0.015d	0.0487±0.0026f	0.0139±0.0010d	0.0110±0.0007e	10.53±0.51e	13.43±0.65f

Data are the means of three replicates (mean ± SD). Values followed by the different letters indicate significant differences followed by Duncan's multiple range test ($p<0.05$) for each cultivar at different metal application treatments.
